# New drug candidates for treatment of atypical meningiomas: An integrated approach using gene expression signatures for drug repurposing

**DOI:** 10.1371/journal.pone.0194701

**Published:** 2018-03-20

**Authors:** Zsolt Zador, Andrew T. King, Nophar Geifman

**Affiliations:** 1 Department of Neurosurgery, Salford Royal NHS Foundation Trust, Salford, United Kingdom; 2 Institute of Cardiovascular Sciences, Centre for Vascular and Stroke Research, University of Manchester, Manchester, United Kingdom; 3 Division of Neurosurgery, Department of Surgery, St. Michaels Hospital, University of Toronto, Toronto, ON, Canada; 4 Centre for Health Informatics, Division of Informatics, Imaging & Data Sciences, University of Manchester, Manchester, United Kingdom; 5 The Manchester Molecular Pathology Innovation Centre, University of Manchester, Manchester, United Kingdom; Northwestren University, UNITED STATES

## Abstract

**Background:**

Atypical meningiomas are common central nervous system neoplasms with high recurrence rate and poorer prognosis compared to their grade I counterparts. Surgical excision and radiotherapy remains the mainstay therapy but medical treatments are limited. We explore new drug candidates using computational drug repurposing based on the gene expression signature of atypical meningioma tissue with subsequent analysis of drug-generated expression profiles. We further explore possible mechanisms of action for the identified drug candidates using ingenuity pathway analysis (IPA).

**Methods:**

We extracted gene expression profiles for atypical meningiomas (12 samples) and normal meningeal tissue (4 samples) from the Gene Expression Omnibus, which were then used to generate a gene signature comprising of 281 differentially expressed genes. Drug candidates were explored using both the Board Institute Connectivity Map (cmap) and Library of Integrated Network-Based Cellular Signatures (LINCS). Functional analysis of significant differential gene expression for drug candidates was performed with IPA.

**Results:**

Using our integrated approach, we identified multiple, already licensed, drug candidates such as emetine, verteporfin, phenoxybenzamine and trazodone. Analysis with IPA revealed that these drugs target signal cascades potentially relevant in pathogenesis of meningiomas, particular examples are the effect on ERK by trazodone, MAP kinases by emetine, and YAP-1 protein by verteporfin.

**Conclusion:**

Gene expression profiling and use of drug expression profiles have yielded several plausible drug candidates for treating atypical meningioma, some of which have already been suggested by preceding studies. Although our analyses suggested multiple anti-tumour mechanisms for these drugs, further *in vivo* studies are required for validation.

**Importance of the study:**

To our knowledge this is the first study which combines relatively new, yet established computational techniques to identify additional treatments for a difficult to manage cerebral neoplasm. Beyond proposing already approved drug candidates in the management of atypical meningioma the study highlights the promise held by computational techniques in improving our management strategies.

## Introduction

Meningiomas are the most frequently encountered brain tumours in adults with an incidence of 1–8 per 100,000 person per year [1]. They constitute approximately one third of all central nervous system neoplasms and are subdivided into 3 tiers based on histopathological properties by the World Health Organization grading. The majority (65–80%) are benign slow growing lesions (WHO grade I) with an 80–90% chance of 5-year disease-free survival post treatment [2]. The remaining subtypes are atypical (WHO grade II) and malignant meningiomas (WHO grade III), which carry more aggressive characteristics and consequently greater morbidity and mortality. The most common of these subtypes is atypical meningiomas, which were initially thought to constitute only 5% of all cases. However with the introduction of the 2000 and 2007 WHO criteria, atypical meningiomas apparently put out 20–35% of all cases [3] with WHO grade III meningiomas representing less than 3% of new diagnosis [4].

Current management of atypical meningiomas is maximal safe surgical excision, yielding a 10-year disease specific survival rate of 69% after first recurrence [5]. Use of radiotherapy for atypical meningiomas currently varies across centers and is mostly applied in cases where only subtotal resection could be achieved or surgery was not feasible. Review of evidence from between 1966–2010 demonstrated improvement of local control with adjuvant radiotherapy [6] in particular with subtotal resection. However even with combined surgical treatment and radiotherapy median 5-year progression free survival rate was 54.2% and the mean 5-year overall survival was 67.5% [6].

These outcomes have upheld the need for additional treatment modalities such as chemotherapy. Several drug trials have targeted key mechanisms of oncogenesis in recurrent/inoperable meningiomas such as cell replication (cytotoxic agents), hormonal mechanisms (progesterone antagonists), aberrant cell signaling (e.g. growth factor and downstream signaling mechanisms) and angiogenesis (VEGF inhibitors). Results were mixed overall, only some candidates were promising in a small case series [7], and high volume studies to solidify efficacy and safety profiles are still lacking. As the treatment of atypical meningiomas remains problematic, the desire persists to identify further drug candidates to improve outcomes.

Drug repurposing allows the reapplication of existing medical therapies to alternative diseases either by screening entire drug libraries [8] or based on similarities of drug and disease signatures [9]. There are several advantages of drug repurposing over conventional de-novo drug development. By screening already approved candidates it allows for bypassing the safety profiling stage translating into lower cost and better time efficiency. Therefore the average 15-year and over $1 billion associated with bringing a new drug onto the market can be substantially reduced [10]. Computational techniques carry the benefit of hypothesis generation, i.e. the identification of drug-disease pairs, which can then inform systemic testing of candidate compounds. Computational drug repositioning in the past has yielded several new candidates such as terbutaline sulfate for amyotrophic lateral sclerosis [11] or the anticonvulsant topiramate in the treatment of inflammatory bowel disease [12]. Disease signatures may be derived from a variety sources including biomedical literature, protein interactions, chemoinformatics or genetic data. Linking disease-drug profiles based on genetic information is now one of the most well-established modalities of drug repurposing, and which is freely achievable through several online resources. The first step of this technique is to acquire a specific signature constituted of genes that are up- or down regulated in the disease state. The disease is then paired with drug candidates based on similarities shared between the disease-specific signature and the expression profile various drugs induce in cultured human cells. Examples for such catalogues of transcriptomic responses to drugs include the Connectivity Map (cmap), establishing the effects of 7,000 expression profiles representing 1,309 compounds [13]. Another similar database is the NIH Library of Integrated Network-Based Cellular Signatures (LINCS), which tests 2,915 drugs in 9 cell lines and a further 12,761 small molecules with lighter coverage in a variety of 37 cell lines [14]. In the current study we applied the gene expression signature of atypical meningiomas [15] to cmap and LINCS to propose new drug candidates for augmenting our current management of this complex and intractable disease.

## Methods

### Disease gene expression data analysis

Disease expression data was obtained from the NCBI Gene Expression Omnibus (GEO) [16,17]. Our analyses focused on data obtained from study GSE4329015. In brief, this study measured gene expression levels from 47 meningioma tumour samples (of which 12 were atypical meningioma tumours) and from 4 normal meninges samples. Raw data files from this study were downloaded and normalized using Robust Multi-array Average (RMA) implemented in GenePattern’s [18] ExpressionFileCreator module. Functional analysis of atypical meningioma vs. normal meninges was conducted using Gene Set Enrichment Analysis (GSEA) [19] implemented in GenePattern [18].

Differential gene expression analysis between atypical meningioma and normal meninges was conducted using the ComparativeMarkerSlection module. To select significant differentially expressed genes for our disease gene signature we applied a fold-change threshold of >3, p-value <0.001 and a false discovery rate (FDR) threshold of <0.05.

### Analysis of drug-induced gene expression signatures

**Fig 1:**
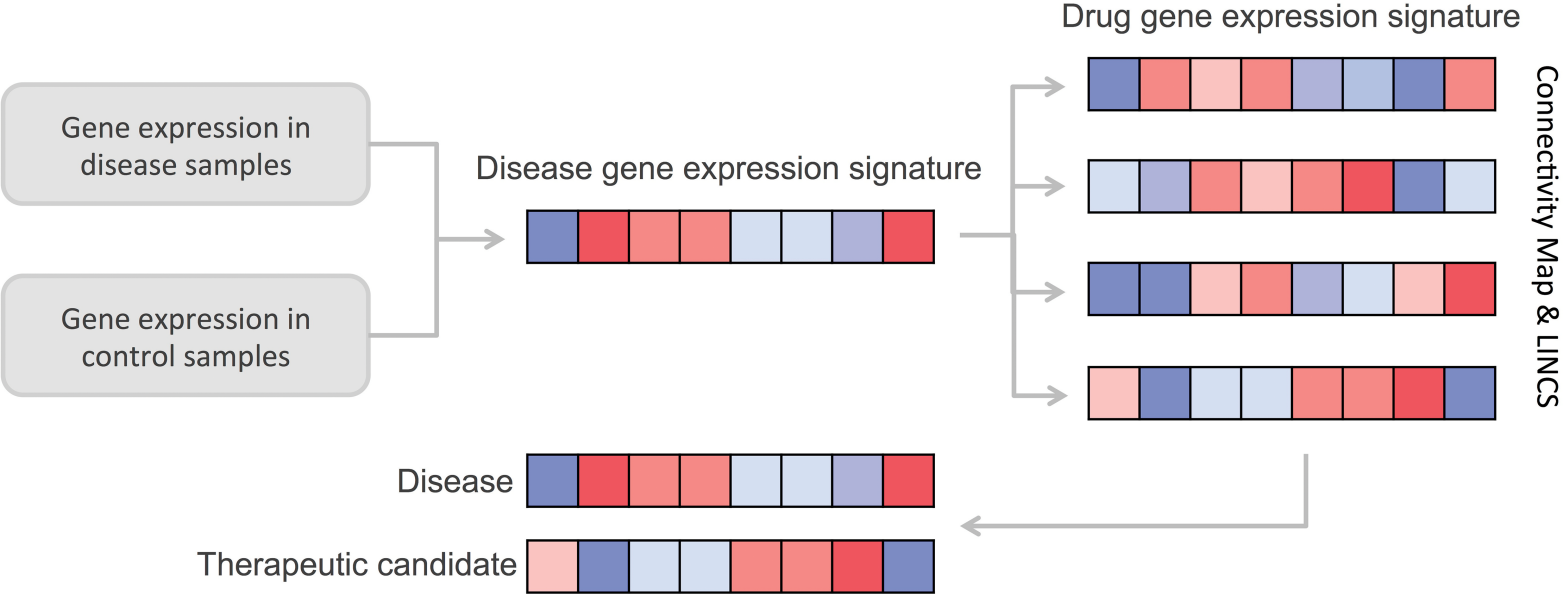
Analysis pipeline. In the first step gene expression values are compared between disease and control sample sets, resulting in a disease-derived gene expression signature. Next the disease expression signature is used to query databases of signatures associated with a variety of compounds. Compounds demonstrating an expression signature oppositional (anti-correlating) to that of the disease are suggested as possible therapeutic candidates.

Disease-specific gene expression signatures (generated as described above) were used to query against gene expression profiles of drugs obtained from the Connectivity Map [13] and Library of Integrated Network-Based Cellular Signatures (LINCS) [20] (Fig 1). In brief, the Connectivity Map (cmap) is a collection of gene expression data from cultured human cells treated with bioactive small molecules and contains more than 7,000 expression profiles representing 1,309 compounds. The LINCS project includes assay results from cultured and primary human cells treated with bioactive small molecules, ligands such as growth factors and cytokines, or genetic perturbations. The L1000 dataset of LINCS includes 2,915 drugs in 9 cell lines and a further 12,761 small molecules with lighter coverage in a variety of 37 cell lines. In addition to making these data available, both cmap and L1000, include a simple pattern-matching algorithm that uses common changes in gene-expression to enable the discovery of functional connections between drugs, genes and diseases [13,21]. In both cases, the systems generate a list of signatures rank ordered by the strength of the match to the query. Each signature is given a score ranging from -1 to 1, were 1 signifies a correlating differential expression pattern and a score of -1 signifies a gene expression pattern that is oppositional to the disease-specific gene expression pattern. Molecules which induce a gene expression signature that is oppositional to that of the disease represent potential therapeutic candidates (Fig 1).

### Pathway analysis

The links between each of the drug candidates and the disease-derived gene signature was further explored using Ingenuity Pathways Analysis (IPA) [22]. For each of the candidate drugs, a network was generated to illustrate the links between the drug and genes differentially expressed in atypical meningioma (our disease gene signature). In these networks, genes and drugs candidates are represented as nodes, and are connected by an edge if there is at least one association between the two (based on IPA’s interactions database).

## Results

### Differential gene expression in atypical meningioma

The results of the original gene expression experiment can be found in reference [23]. To interpret the biological significance of atypical meningioma gene expression levels we assessed its enrichment with KEGG23 pathways and Gene Ontology (GO) terms [24]. Using GSEA, several KEGG pathways suggestive of neoplastic processes were found to be significantly up-regulated in atypical meningioma (Table 1). These included DNA excision repair, RNA polymerase, pathways related to thyroid cancer and endometrial cancer. In contrast, the only pathway found to be significantly down-regulated was the NOD-like receptor-signaling pathway. This was somewhat unexpected as this pathway is primary associated with inflammatory processes in particular chronic conditions such as Crohn’s disease or Blau syndrome. Analysis of Gene Ontology terms significantly associated with atypical meningioma confirmed up-regulation of categories intuitive of neoplasia such as Insulin receptor signaling pathway, transmembrane receptor protein tyrosine kinase signaling pathway, and base and nucleotide excision repair (Table 1). Several GO terms related to cell maturation and development were down-regulated in atypical menigiomas, in keeping with a neoplastic process.

**Table 1:** Interpretation of gene expression levels.

KEGG Pathways			
Up-regulated		Down-regulated	
**Pathway**	**P-value**	**Pathway**	**P-value**
Base excision repair	0	NOD like receptor signaling pathway	0.0325
Thyroid cancer	0		
RNA polymerase	0.0055		
Endometrial cancer	0.0068		
N glycan biosynthesis	0.0132		
Adherens junction	0.0138		
Pyrimidine metabolism	0.0268		
Nucleotide excision repair	0.0323		
Non small cell lung cancer	0.0337		
Galactose metabolism	0.0491		
Gene Ontology Biological Processes			
Up-regulated		Down-regulated	
**Process**	**P-value**	**Process**	**P-value**
Positive regulation of phosphate metabolic process	0.0074	Cell maturation	0.0081
Insulin receptor signaling pathway	0.0087	Developmental maturation	0.0084
RNA export from nucleus	0.0111	Cell migration	0.0099
Transmembrane receptor protein tyrosine kinase signaling pathway	0.0112	Negative regulation of map kinase activity	0.0111
Base excision repair	0.0123	Muscle development	0.0143
Cellular protein complex assembly	0.0133	Rhythmic process	0.0195
Transcription initiation from RNA polymerase ii promoter	0.0134	Microtubule based movement	0.0196
Protein amino acid n linked glycosylation	0.0136	Regulation of biological quality	0.0197
Nuclear export	0.0152	Cell development	0.0203
Protein complex assembly	0.0159	Synaptic transmission	0.0209
Regulation of protein modification process	0.0167	Nervous system development	0.0217
Mitochondrion organization and biogenesis	0.0206	Regulation of action potential	0.0219
Nucleotide excision repair	0.0223	Muscle cell differentiation	0.0225
Stress activated protein kinase signaling pathway	0.0248	Myoblast differentiation	0.0228
Positive regulation of protein modification process	0.0261	Cytoskeleton dependent intracellular transport	0.0264
Carbohydrate metabolic process	0.0268	Negative regulation of cell differentiation	0.0297
Positive regulation of protein metabolic process	0.0269	Behavior	0.0309
Macromolecular complex assembly	0.0270	Protein homooligomerization	0.0325
Cellular component assembly	0.0336	Transmission of nerve impulse	0.035
JNK cascade	0.0358	Central nervous system development	0.0352
DNA repair	0.0361	Regulation of g protein coupled receptor protein signaling pathway	0.0374
Carbohydrate catabolic process	0.0363	Skeletal muscle development	0.0384
Cellular carbohydrate catabolic process	0.0363	Cell proliferation go 0008283	0.0386
Transcription initiation	0.0401	Locomotory behavior	0.0493
Interaction with host	0.0449		
Nucleobasenucleosidenucleotide and nucleic acid transport	0.0455		
Positive regulation of metabolic process	0.0465		
Regulation of protein amino acid phosphorylation	0.0479		
Golgi vesicle transport	0.0486		
Glycoprotein metabolic process	0.0493		
Positive regulation of phosphorylation	0.0495		

Interpretation of gene expression levels using enrichment analysis with KEGG pathways and Gene Ontology (GO) terms.

### A gene signature for atypical meningioma

**Fig 2:**
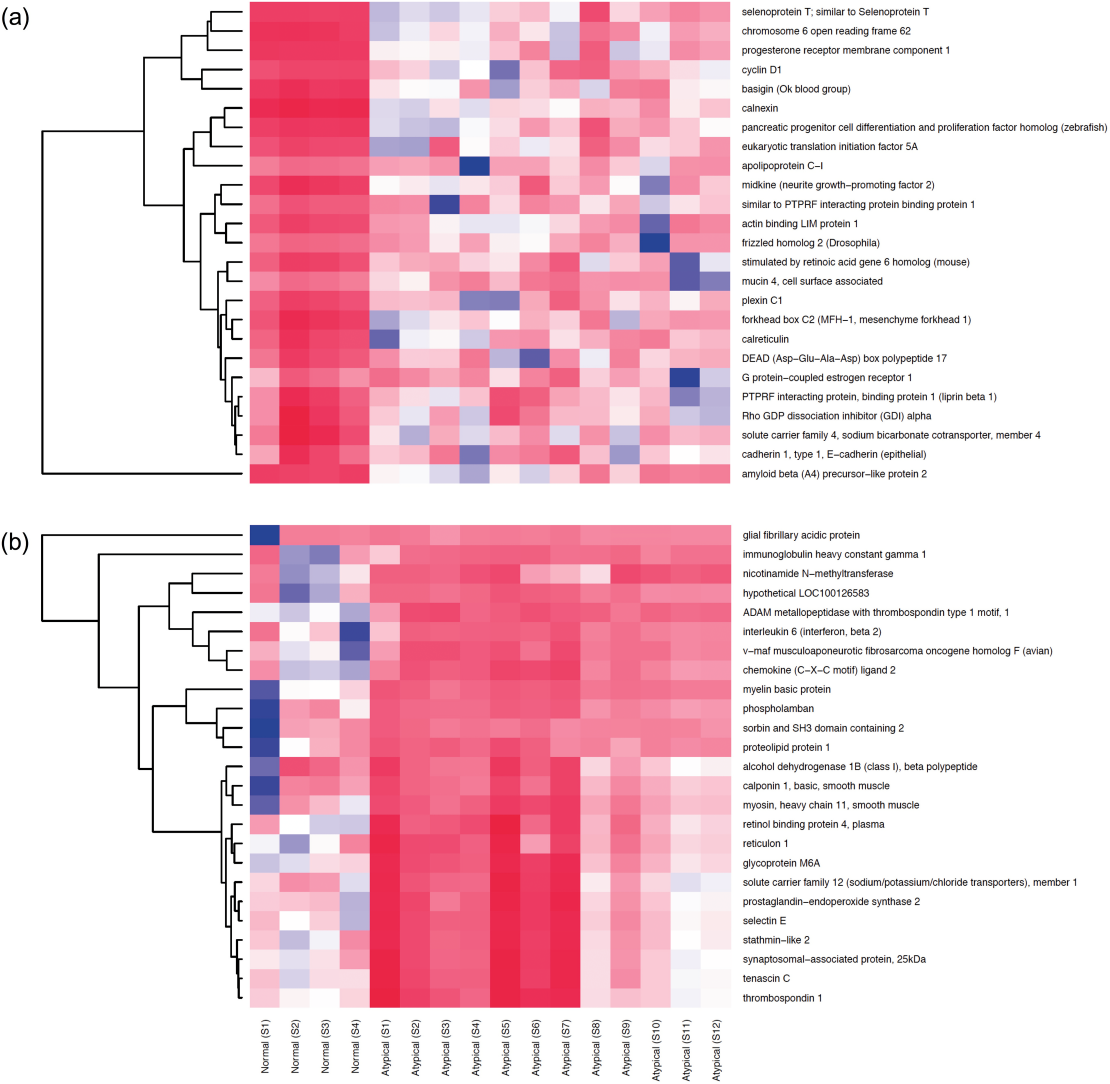
Gene expression heatmaps for most differentially expressed genes in atypical meningioma. (a) Top 25 up-regulated genes in atypical meningioma in comparison to normal meninges. (b) Top 25 down-regulated genes in atypical meningioma in comparison to normal meninges.

We next derived a gene expression signature for atypical meningiomas by comparing gene expression levels between atypical meningioma tumour samples (n = 12) and normal meninges samples (n = 4). After applying a fold-change threshold of >3, p-value<0.001 and an FDR threshold of <0.05, 42 transcripts were found to be up-regulated while 239 were down-regulated in atypical meningioma relative to normal samples (Fig 2). Up-regulated genes included cyclin D1, G protein-coupled estrogen receptor 1, neurite growth-promoting factor 2, amyloid beta (A4) precursor-like protein 2, and calreticulin. While down-regulated genes included tumour suppressor genes (deleted in liver cancer 1), extracellular matrix components (tenascisn C, lumican, decorin, sarcoglycan, epsilon), cell adhesion molecules (platelet and endothelial cell adhesion molecule 1[PECAM-1], Intercellular adhesion molecule 2 [ICAM-2], E selectin) and genes of structural molecules (desmin, actin, tubulin, myosin heavy chain).

### Drug repurposing for atypical meningioma

The gene expression signature generated for atypical meningioma was used to query two repositories for perturbation-induced expression signatures. These queries resulted in a list of bioreactive small-molecules that demonstrate gene expression signatures that are anti-correlated to that of atypical meningioma and therefore represent potential therapeutic candidates (Table 2).

**Table 2:** Top drug repurposing candidates from cmap and LINCS.

Drug name	C-map score	LINCS score
verteporfin	-0.749	-0.4658
emetine	-0.721	-0.6047
phenoxybenzamine	-0.708	-0.3464
trazodone	-0.601	-0.3759
omeprazole	-0.616	-0.3973
sulconazole	-0.610	-0.4093
8-azaguanine	-0.603	-
azacitidine	0.638	-0.6268
IL1	-	-0.6141
cercosporin	-	-0.6042

Only drug candidates with a connectivity score of <-0.6 (from at least one data resource) are displayed, excluding non-drug small molecules; for cmap candidates, we only included those with a p value<0.05. For cmap scores, the arithmetic mean of the connectivity scores is given for each of the listed candidates. For LINCS scores, the best (lowest) score is given for each of the listed candidates.

### Drug candidate pathway analysis

**Fig 3:**
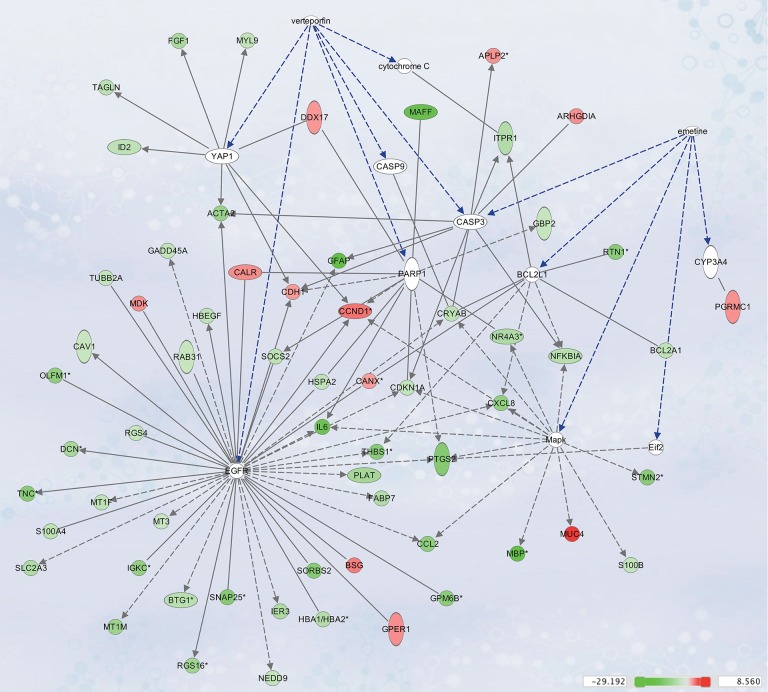
IPA interaction network for verteporfin and emetine, and differentially expressed genes from our atypical meningioma gene signature. Genes and drugs candidates are represented as nodes, and are coloured based on fold-change of expression values, ranging from green (down-regulated in atypical meningioma) to red (up-regulated in atypical meningioma). Nodes in white represent genes that were not included in our atypical meningioma gene signature but serve as a link in the network to the candidate drugs. A line connects two nodes if there is any known relationship between the two; solid lines represent direct interactions while dotted lines represent indirect interactions.

We used Ingenuity Pathway Analysis for the highest-ranking drug candidates to assess their link to the differential gene expression profile and pathways. The association between the top two drug candidates, verteporfin and emetine, and genes from our atypical meningioma gene signature are depicted in Fig 3. Interactions from the pathway analysis showed verteporfin and emetin’s direct/indirect interactions with cascades relevant to apoptosis (caspase 3, 9, B-cell lymphoma 2 [BLC-2], cytochrome c) or cell proliferation (Epidermal Growth Factor Receptor [EGFR], and Mitogen Activated Protein Kinase [MAPK]). Interestingly, both emetine and verteporfin interacted with caspase 3.

## Discussion

Multiple, successful new therapies have been identified in the past using drug repurposing based on differential gene expression profiles. Examples include amyotrophic lateral sclerosis [11] and inflammatory bowel disease [12]. The strategies applied to verify drug candidates in these studies were based on electronic patient records [11] or experimental studies in animal models [12], respectively. Atypical meningiomas are difficult to manage due to high recurrence rate, and treatment modalities are limited to surgical excision and radiotherapy. In the current paper we take a fully computational approach to support potential effects of drug candidates by bio-functional analysis in IPA. Each of the top three drug candidates were found to interact with at least one of the cascades previously proposed in the pathogenesis of meningioma.

### Verteprofin

Verteporfin ranked as the best candidate based on its connectivity score in both cmap and LINCS; it is currently used in ophthalmology as a photosensitizer in the treatment of secondary choroidal neovascularization and other conditions including choroidal hemangiomas [25]. Our pathway analysis showed its indirect interactions with transcription factors relevant to cell differentiation, apoptosis and ontogenesis such as yes-associated protein 1 [YAP1], cytochrome C, Poly [ADP-ribose] polymerase 1 (PARP-1), caspase 3, caspase 9, and EGFR. The relevance of these transcription factors in meningioma biology is well traceable in the literature.

YAP1 overexpression can result in increased contact independent cell proliferation [26]. Subsequently YAP1 was found to express in all grades of meningiomas and its deletion caused impaired cell proliferation and migration in vitro, whereas overexpression translates to proliferation and anchorage independent cell growth [27]. Recent results demonstrated that verteporfin interferes with the TEAD (TEA domain family member)-YAP pathway [28], which has been suggested to drive the neoplastic transformation of arachnoid cap cells and promote meiningioma progression. Additionally, in vitro evidence showed inhibition of meningioma growth together with an increased sensitivity to irradiation post verteporfin treatment [29].

EGFR has been previously identified as a promising therapeutic target in non-small cell lung tumours, with treatment effect linked to point mutations of the tyrosine kinase domain [30]. EGFR is expressed in 50–80% of meningiomas [31], and activation of the EGFR signal was shown to stimulate meningioma proliferation in vitro [32], further suggesting it as a potential treatment target. However in a recent phase II trial consisting of twenty-five patients, EGFR inhibitors gefitinib and erlotinib did not show significant activity against atypical meningioma [33]. Furthermore, evidence suggests a lack of mutations in the thyrosine kinase domain of the receptor previously linked to treatment response [31]. Although the role of EGFR in meningioma treatment remains uncertain, our results suggest verteporfin may offer a new therapeutic action via this pathway.

Another potentially anti-neoplastic action of verteporfin proposed by IPA is the effect on cytochrome-c. The cytoplasmic release of cytochrome c is a key factor in mitochondria dependent apoptosis. This mechanism has been demonstrated in meningioma cell lines by modulating cytochrome-c release through the N-Myc Downstream Regulated Gene 4 (NDRG4). Silencing of NDRG4 translated to an up-regulation of p53 and subsequent release of cytochrome c with significantly reduced proliferation rates in meningioma cell cultures [34]. These findings further support the therapeutic action of verteporfin through cytochrome c in meningiomas.

Other potential targets for verteporfin include: PARP-1, which is involved in single strand DNA repairs and was recently demonstrated to have highest expression levels in grade II subtypes meningiomas [35] and the pro-apoptotic protein caspase-3 shown to correlate with the histological grade of meningioma, cell proliferation index and mitotic count [36]. Furthermore the presence of active Caspase 3 fragments have been demonstrated in atypical meningiomas [37]. Verteporfin has been shown to induce apoptotic cell death in HeLa cells via induction of caspase 3 [38], however this mechanism has not been demonstrated in meningioma cells. While these findings need to be interpreted in the context of caspase regulators, the presence of caspase component (including caspase 3) in atypical meningioma support the hypothesis of verteporfin effect.

### Emetine

A second proposed candidate, emetine, is a naturally occurring substance extracted from the syrup of ipecac and has a long history of use for the treatment of amoebiasis dating back to the 1500s. It is currently the drug of choice to induce emesis after ingestion of potential toxins [39]. Emetine has been available as an over the counter medication since 1966 in the United States with over 50,000 doses given per year according to poison control center records [40]. On a cellular level it has been identified as an inhibitor of translation [41] and has been widely used suppressor of protein synthesis in cellular models [42]. Glioblastoma multiforme cell lines pretreated with emetine and transplanted intraparenchymaly demonstrated reduced tumour growth in mouse models [43] supporting emetine as a promising candidate for treating atypical meningioma.

Our IPA analysis revealed that emetine interacts with BLC-2, MAPK, Eukaryotic translation initiation factor 2 (eIF2) and Cytochrome P450 3A4 (CYP3A4) proteins. Similar to verteporfin it had an interaction with caspase 3. The anti-apoptotic protein BLC-2 has been detected in meningiomas. Although not associated with clinical outcome it was more abundantly expressed in atypical variants [44]. MAPK activation has been implicated in the pathogenesis of meningiomas [45] and its inhibition resulted in slowed cell growth and increased apoptosis in malignant meningioma cultures [46] at the expense of increased recurrence. A key regulator of cell proliferation, eIF2, has low expression levels in meninges but is increased in atypical meningioma tissue [47]. CYP3A4 is a member of the cytochrome P450 family (CYP) and metabolises neuroactive steroids such as testosterone and estradiol in cerebral tissue [48]. So far it has not been implicated in meningioma pathogenesis, however its involvement in testosterone and estradiol metabolism may suggest its relevance as a significant portion of meningiomas are thought to be “hormone fed” [49].

### Phenoxybenzamine

Phenoxybenzamine is a non-selective alpha-blocker mainly used for its antihypertensive effects in the setting of pheochromocytoma. It has been identified as a small molecular inhibitor of glioblastoma cell viability and invasion in vivo [50]. This effect was suggested to be independent of its alpha-antagonist function and attributed to its inhibitory effect on the EGFR pathway [51]. As discussed above, several lines of evidence support the role of EGFR signaling pathway in the pathogenesis of meningiomas.

Phenoxybenzamine also binds and inhibits calmodulin [52], a ubiquitous calcium binding protein, which promotes neoplasia [53] by enhancing cell proliferation, tumour growth, angiogenesis and metastasis [54]. Although calmodulin expression is documented in meningiomas [55] its contribution to meningioma pathogenesis remains to be established. Potential anti-neoplastic effects of phenoxybenzamine may also occur through prolactin or glucocorticoid related pathways in meningiomas [52,56].

Other potential candidates include trazodone, omeprazole, sulconazole and 8-azaguanine, however these were associated with lower scores or supported by evidence from only one of the drug-signature resources we searched and are therefore less likely to be of interest. An additional drug that was identified by our approach is azacitidine, however, the scores from cmap and LINCS were contradicting. While the drug’s gene expression signature from LINCS was oppositional to that associated with atypical meningioma, suggesting it may be a potential therapeutic candidate, the gene signature from cmap had the same directionality as the disease. The most likely reason for the disparity in these outputs is the underlying differences between cmap and LINCS. Such differences include the number of genes examined for each of the tested drugs (1000 in LINCS and ~20,000 in cmap), the number of drugs for which a signature was generated (up to 15,676 in LINCS and 1,309 in cmap) and the specific cell lines used to produce the drug-associated gene expression signatures. This mismatch between the two resources illustrates a potential pitfall in such analyses, where the results are somewhat dependable on how the drug gene expression signatures were generated. By conducting repurposing analyses using more than one data resource, and by integrating the results, as was performed here, the confidence in concordant potential candidates increases.

### Study limitations

Our study derives drug candidates from a single genetic dataset. Although these candidates scored high on the c-map and LINCS matching algorithms it was still desirable to increase the power of the analysis by including additional datasets. We have explored this option and identified multiple datasets describing gene expression profiles for human atypical meningioma tissue [15,57–63]. On review of these datasets we have identified the following difficulties with integrating them into our analysis: 1) the high throughput platforms used in these studies did not interface with the c-map or LINCS 2) Neither of the additional studies had normal meninges as controls and inclusion of atypical meningioma data alone would render the combined dataset unbalanced from our purposes 3) finally there was a concern regarding the batch effect caused by the inclusion of additional (unbalanced) studies, particularly given the lack of “normal” meningeal tissue in these dataset. The batch effect is a collective term used to describe sources of variation other then the biological effect of interest (differences in handling, techniques and processing for example). It is an increasingly recognized hurdle in data analytics and has been a topic of recent discussions (reviewed by Goh et al [64]) and also suggested as one of the newly emerging challenges of the field. Batch effect correction algorithms are being developed to tackle the problem however there is limited knowledge on their reliability and they are particularly vulnerable to increased false positive or false negative rates in unbalanced datasets [65,66].

## Conclusion

Our analysis has successfully identified a series of small molecules as potential drug candidates to expand the chemotherapeutic treatment of atypical meningiomas. Ingenuity pathway analysis provided further insight into the mechanism of action for the drug candidates proposed by the connectivity map and LINCS. While our analyses have uncovered some highly plausible drug candidates for the treatment of atypical meningiomas, this approach is hypothesis-generating, and experimental validation of our results is necessary before clinical translation.
